# Transpulmonary pressure in SARS-CoV-2-associated acute respiratory distress syndrome: a single-center observational study

**DOI:** 10.1186/s13054-020-03129-5

**Published:** 2020-07-09

**Authors:** Severin Ramin, Jonathan Charbit, Geoffrey Dagod, Mehdi Girard, Samir Jaber, Xavier Capdevila

**Affiliations:** 1 Anesthesiology and Intensive Care, Anesthesia and Critical Care Department A, Lapeyronie Teaching Hospital, Montpellier Cedex 5, France; 2 Département d’Anesthésie Réanimation Lapeyronie, Hôpital Lapeyronie, 371 Avenue du Doyen G. Giraud, 34090 Montpellier, France; 3 Anesthesiology and Intensive Care, Anesthesia and Critical Care Department B, Saint Eloi Teaching Hospital, PhyMedExp, University of Montpellier, INSERM U1046, CNRS, UMR 9214, 80 Avenue Augustin Fliche, 34295 Montpellier Cedex 5, France; 4 Département d’Anesthésie Réanimation Saint-Eloi, Hôpital Saint-Eloi, 80 Avenue Augustin Fliche, 34090 Montpellier, France

Dear Editor,

Gattinoni et al. [1] have recently described that the ARDS related to severe acute respiratory syndrome coronavirus 2 (SARS-CoV-2) was not a “typical” ARDS. Patients indeed presented a significant hypoxemia, which was surprisingly associated with a high compliance of the respiratory system. The cornerstone of current treatment is the use of “lung protective” ventilation strategy with especially maintaining sufficiently high positive end-expiratory pressures (PEEP). However, high levels of PEEP may lead to lung overdistension associated with an increase of alveolar dead space and an alteration of gas exchanges. The airway pressures commonly monitored on respirators do not reliably reflect the impact of pressures on the lung parenchyma. In contrast, transpulmonary pressures allow to highlight directly lung overdistension risk and lung properties. In order to better know this new kind of ARDS, transpulmonary pressures’ assessment seems to be essential [2].

We wish to report the preliminary findings of a prospective monocentric physiological work, which is approved by the institutional ethics review board of the Montpellier University Hospital, France (IRB ID: 202000432). All consecutive ARDS adult patients with confirmed Covid-19 admitted in our critical care unit are included if they received invasive mechanical. Management of patients followed international recommendations. PEEP was set according to the low PEEP arm of the PEEP/F_i_O_2_ table used in previous trials. Esophageal pressures were recorded with Cooper Surgical® device.

Sixteen patients were assessed (body mass index 29 [range 26–31] kg/m^2^, age 70 years [range 61–72 years]). The median worst value of PaO_2_/F_i_O_2_ ratio was 144 mmHg [range 138–149 mmHg]. Baseline of patient characteristics may be observed in the Table 1. Six patients received at least one session of prone positioning. One of them also benefited from VV-ECMO. We observed that median transpulmonary end-expiratory pressure was positive during mechanical ventilation (2 cm H_2_O at D1 [range − 1–3 cm H_2_O]) and median transpulmonary elastance (E_L_) remained low at day 1 (19 cm H_2_O/L [range 8–20 cm H_2_O/L]) and during the first week. All our findings are shown in Fig. 1.

**Table 1:** Baseline characteristics of the patients and respiratory mechanics (*n* = 16)

**Characteristics**	
Male, *n* (%)	11 (69)
Age (years)	70 (61–72)
Body mass index (kg/m^2^)	29 (26–31)
Organ failure at baseline (SOFA), *n* (%)	
Hemodynamic	12 (75)
Renal	4 (25)
Hepatic	2 (13)
Hematological	1 (6)
Arterial blood gas	
PaO2/FiO2 ratio	170 (150–208)
pH	7.43 (7.38–7.47)
PaO2 (mmHg)	71 (62–89)
PaCO2 (mmHg)	40 (37–45)
Lactates (mmol/l)	1.2 (1–1.4)
Radiologic characteristics, *n* (%)	
Bilateral pneumonia	4 (25)
Multiple mottling and ground-glass opacity	12 (75)
Time between admission and intubation (day)	2 (2–3)
**Respiratory mechanics at day 1**	
Tidal volume (ml/kg PBW)	7 (6.6–7.3)
Respiratory rate (beats/min)	22 (20–24)
PEEPtot,rs (cm H_2_O)	10 (10–13)
PEEPtot,es (cm H_2_O)	8 (8–12)
PEEPtot,L (cm H_2_O)	2 (−1–3)
Pplat,rs (cm H_2_O)	19 (18–22)
Pplat,es (cm H_2_O)	13 (11–16)
Pplat,L (cm H_2_O)	6 (6–11)
DP,rs (cm H_2_O)	9 (6–12)
DP,cw (cm H_2_O)	5 (3–5)
DP,L (cm H_2_O)	4 (2–9)
Est,rs (cm H_2_O/L)	23 (18–30)
Est,cw (cm H_2_O/L)	4 (4–10)
Est,L (cm H_2_O/L)	19 (8–20)

Results are expressed as median (IQR) or as number of patients (percentage) as appropriate

*PBW*, predicted body weight; *SOFA*, sepsis-related organ failure; *PEEPtot,rs*, static end-expiratory pressure of the respiratory system; *PEEPtot,es*, static end-expiratory esophageal pressure; *PEEPtot,L*, static end-expiratory transpulmonary pressure; *Pplat,rs*, static end-inspiratory pressure of the respiratory system; *Pplat,es*, static end-inspiratory esophageal pressure; *Pplat,L*, static end-inspiratory transpulmonary pressure; *DP,rs*, *DP,cw*, *DP,L*, driving pressure of respiratory system, chest wall, and lung, respectively; *Est,rs*, *Est,cw*, *Est,L*, static elastance of respiratory system, chest wall, and lung, respectively

**Fig. 1:**
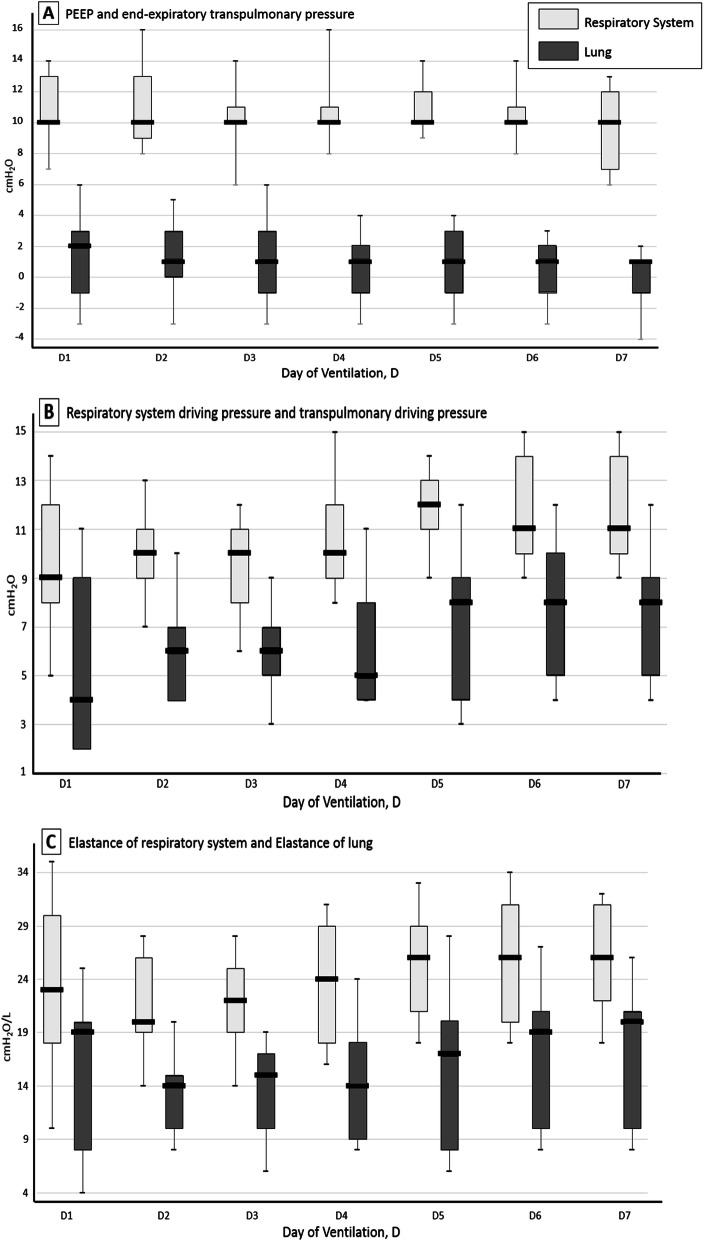
Respiratory physiological measures during mechanical ventilation. Boxes represent median and interquartile range. The number of patients with available respiratory physiological data decreases over successive study days due to deaths and discontinuation of invasive mechanical ventilation. Transpulmonary pressure (PL) equals respiratory system pressure minus esophageal pressure. Respiratory system driving pressure equals plateau pressure minus positive end expiratory pressure (PEEP). Transpulmonary driving pressure equals end-inspiratory PL minus end-expiratory PL

Many reports have highlighted specificities of SARS-CoV-2, particularly that the elastance of respiratory system (E_RS_) was slightly altered. Driving pressures of the respiratory system are therefore quite low in these patients. Unsurprisingly, our study showed that the E_**L**_ was not elevated in the first days of mechanical ventilation. The main finding of the present work is that end-expiratory transpulmonary pressure remained positive in most patients with the use of moderate PEEP (8–13 cm H_2_O). To summarize, our analysis is in agreement with the descriptions of Gattinoni et al. [3]. Most of these patients present indeed low E_RS_, low ventilation to perfusion ratio (VA/Q), and low lung recruitability due to the low amount of non-aerated tissue (*L profile*). Hypoxemia and intrapulmonary shunt might thus be better explained by dysregulation of pulmonary perfusion and by alteration of hypoxic vasoconstriction. This would justify the use of moderate levels of PEEP to limit alveolar dead space and optimize the CO_2_ removal. Gattinoni et al. [4] also described that 20–30% of patients presented a delayed aggravation with more usual pulmonary parameters: high E_RS_, high right-to-left shunt, high lung recruitability (*H profile*). Constitution of atelectasis or bacterial overinfection is associated with *H profile*. This is observable in our series since a part of patients experienced an increase of E_RS_ as E_L_ (Fig. 1c). For the latter, elevated PEEP seems to be more legitimate [5]. To conclude, identification of respiratory phenotype seems therefore essential in ventilated SARS-CoV-2 patients to determine optimal mechanical ventilation strategy. Our observations support the concept of using low PEEP in a large part of SARS-CoV-2 patients.

## Data Availability

Not applicable.
